# Genome-Wide CRISPR/Cas9 Library Screening Identified that DUSP4 Deficiency Induces Lenvatinib Resistance in Hepatocellular Carcinoma

**DOI:** 10.7150/ijbs.69969

**Published:** 2022-07-04

**Authors:** Shanzhou Huang, Zuyi Ma, Qi Zhou, Aimei Wang, Yuanfeng Gong, Zhenchong Li, Shujie Wang, Qian Yan, Dongping Wang, Baohua Hou, Chuanzhao Zhang

**Affiliations:** 1 Department of General Surgery, Guangdong Provincial People's Hospital, Guangdong Academy of Medical Sciences, Guangzhou 510080, China; 2 Heyuan people's Hospital, Heyuan 517000, China; 3 School of Medicine, South China University of Technology, Guangzhou 510080, China; 4 The Second School of Clinical Medicine, Southern Medical University, Guangzhou, 510515, China; 5 Shantou University of Medical College, Shantou, 515000, China; 6 Department of General Surgery, Hui Ya Hospital of The First Affiliated Hospital of Sun Yat-Sen University; Department of Liver Surgery, The First Affiliated Hospital of Sun Yat-Sen University, Guangzhou, 510080, China; 7 Department of Hepatobiliary Surgery, the Affiliated Cancer Hospital & Institute of Guangzhou Medical University, Guangzhou, 510095, China; 8 Organ Transplant Centre, The First Affiliated Hospital, Sun Yat-sen University, Guangdong Provincial Key Laboratory of Organ Donation and Transplant Immunology, Guangdong Provincial International Cooperation Base of Science and Technology (Organ Transplantation), Guangzhou 510080, China

**Keywords:** Lenvatinib, drug resistance, genome-wide CRISPR/Cas9 library, DUSP4, MAPK/ERK pathway

## Abstract

**Background:** Lenvatinib is in a first-line therapy for advanced hepatocellular carcinoma (HCC). However, drug resistance is one of the principal obstacles for treatment failure. The molecular mechanism of Lenvatinib resistance has not been well investigated.

**Materials and methods:** A genome-wide CRISPR/Cas9 knockout screening system was established and bioinformatic analysis was used to identify critical genes associated with Lenvatinib resistance. Cell proliferation assays, colony formation assays and cell migration assays were performed to investigate the effect of drug resistance associated genes, particularly DUSP4, on cancer cell malignant behavior during Lenvatinib treatment. *In vivo* experiments were conducted by using a xenograft mouse model.

**Results:** We identified six genes that were associated with Lenvatinib resistance in HCC, including DUSP4, CCBL1, DHDH, CNTN2, NOS3 and TNF. DUSP4 was found to be significantly decreased at the mRNA and protein levels in Lenvatinib resistant HCC cells. DUSP4 knockout enhanced HCC cell survival, cell proliferation and migration during Lenvatinib treatment *in vitro* and *in vivo*, accompanied by regulation of p-ERK and p-MEK levels. This finding implied that DUSP4 deficiency induced Lenvatinib resistance. Interestingly, DUSP4 deficiency induced Lenvatinib resistance was abrogated by the MEK inhibitor Selumetinib, implying that MEK phosphorylation and DUSP4-inhibition dependent ERK activation were required for drug resistance. Finally, we found that DUSP4 deficiency was associated with HCC prognosis and response to Lenvatinib based on clinical data.

**Conclusions:** DUSP4 deficiency mediates Lenvatinib resistance by activating MAPK/ERK signaling and combination therapy using Lenvatinib and MEK inhibitors may be a promising therapeutic strategy for overcoming Lenvatinib resistance.

## Introduction

Globally, hepatocellular carcinoma (HCC) is the third leading cause of cancer-associated mortalities, with annual deaths of 740,000 1. The standard curative options for early stage HCC are surgical resection, transplantation and ablation. For HCC patients with chronic disease or in an advanced stage who cannot undergo resection, the oral multikinase inhibitor sorafenib has been used for more than a decade as the only first-line systemic treatment with improved overall survival 2-3. Regorafenib and nivolumab have been approved as systemic treatments for patients with sorafenib-resistant HCC 2-4. To obtain more options for the first-line treatment of advanced HCC, Lenvatinib has been an emerging treatment 5. Lenvatinib is an oral multikinase inhibitor that targets VEGF receptors 1 to 3, FGF receptors 1 to 4, PDGF receptor α, RET, and KIT, and was first approved for the treatment of radioiodine-refractory differentiated thyroid cancer and renal cancer 6-9. A randomized phase III noninferiority trial and the REFLECT trial has demonstrated that the overall survival outcomes for Lenvatinib are comparable to those of sorafenib in untreated advanced HCC 10. However, the clinical efficacy of Lenvatinib treatment is modest since all patients who receive Lenvatinib treatment ultimately progress in their cancer status. The major obstacle for the failure of Lenvatinib treatment might be the development of drug resistance 11. The underlying resistance mechanisms of Lenvatinib and related receptor tyrosine kinase inhibitors have not been elucidated. Studies on the molecular basis of Lenvatinib resistance may aid in the identification of novel targets for rational combinational therapy to overcome Lenvatinib resistance.

High-throughput forward genetic screening approaches are widely used to study the molecular mechanisms underlying specific cellular phenotypes, including drug resistance in malignancies. In particular, RNA interference (RNAi) using a shRNA library or clustered regularly interspaced short palindromic repeats CRISPR-associated nuclease Cas9 (CRISPR/Cas9), is an effective tool to perform loss-of-function screening in a wide range of signaling pathways and biological processes 12-13. In a previous study, MAPK14 was identified as a critical gene involved in Sorafenib resistance in HCC-bearing mice by the pooled shRNA library 12-13. However, RNAi by shRNA only knocks down the expression of target mRNA without eliminating the targeted genes 14-15. To improve on-target knockout efficiency and avoid off-target effects, we performed genome-wide CRISPR/Cas9 knockout screening in HCC cells with or without Lenvatinib treatment to systematically evaluate the underlying mechanisms of Lenvatinib resistance. The screening results demonstrated that dual specificity phosphatase 4 (DUSP4) and MAPK/ERK signaling pathways activation leads to Lenvatinib resistance* in vivo* and *in vitro*. The strategy of formulating a Lenvatinib and Selumetinib combined therapy, could overcome drug resistance, and prolong the survival of HCC patients.

## Materials and Methods

### Screening of the genome-wide CRISPR/Cas9 knockout library

**Figure 1:**
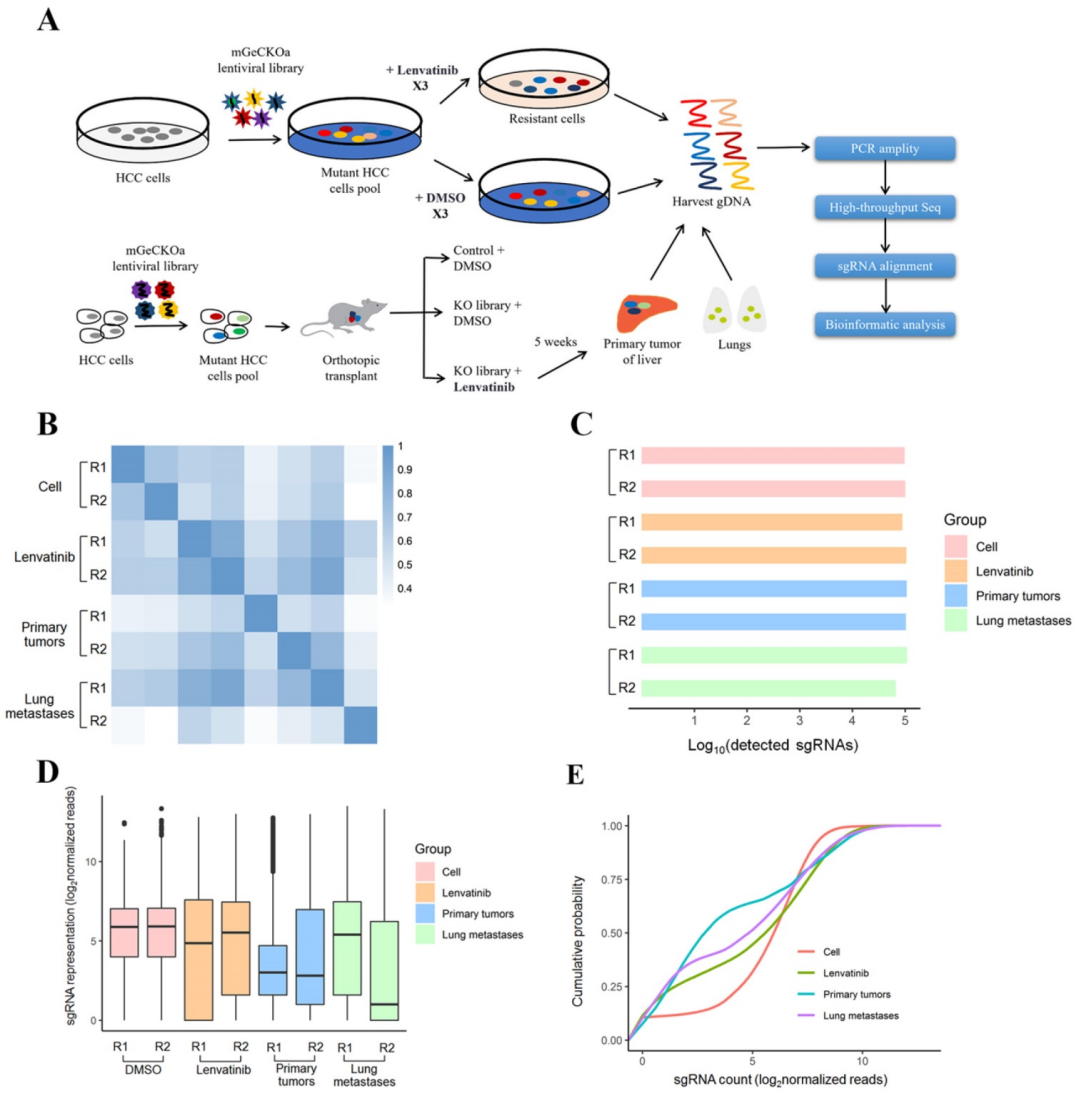
** CRISPR library screening identified crucial genes for Lenvatinib resistance at different stages of tumor growth and metastasis. (A)** Schematic diagram illustrating the workflow of genome-wide CRISPR/Cas9 knockout library screening both *in vivo* and *in vitro*. **(B)** Pearson correlation coefficient of the normalized sgRNA read counts from the hGeCKOa plasmid library. **(C)** Number of unique sgRNAs in the samples. **(D)** Boxplot of the sgRNA normalized read counts for the hGeCKOa plasmid pool. **(E)** Cumulative probability distribution of library sgRNAs.

The human CRISPR Knockout Pooled Library (hGeCKO v2) was used to identify Lenvatinib resistance associated genes in HCC cells. The library was a gift from Feng Zhang (Addgene #1000000049) 16. The workflow of this forward genetic screen at different stages of tumor growth and metastasis is shown in **Figure 1A,** which is based on Zhang's study design 14. First, we established a stable Cas9-expressing HCC cell line by lentiviral transduction of the Cas9 coding sequence. The expression of Cas9 was confirmed by polymerase chain reaction (PCR) (**Fig. S1A**). Then, we transduced HepG2-Cas9 cells with the human genome-scale CRISPR knockout library A (hGeCKOa) and B (hGeCKOb), which contains 65,383 and 58,028 unique sgRNA sequences targeting 19,050 human genes and 1864 miRNAs (3 sgRNAs per gene, 4 sgRNAs per miRNA, and 1000 non-targeting controls), respectively, at a low MOI (~0.3) to ensure effective barcoding of individual cells 15, 17. For the *in vitro* model, the transduced cells were treated with vehicle (DMSO) and Lenvatinib (80 µM) every 6 days for a total of 3 times to generate a mutant cell pool. After treatment, 3 × 10^7^ cells were obtained for genomic DNA extraction to ensure over 400× coverage of the hGeCKO v2 library.

### Sequencing of sgRNA and identification of key genes

The sgRNA sequences of all samples were amplified by PCR from genomic DNA using primers containing adaptor and barcoding sequences. DNA fragments were size selected using agarose gel and sequenced using a 1 x 125 bp run on the HiSeq2500 (Illumina Inc., San Diego, CA, USA). Reads generated from each sample were aligned to the indexed sgRNA sequences using the “very-sensitive-local” option of the Bowtie2 sequence aligner. The sgRNA counts were summarized using htseq; sgRNA counts across all samples were compiled, and differential sgRNA abundance was calculated using DESeq2 17. To map the sgRNA results to the gene level (approximately three gRNAs per gene), we calculated the mean fold change and combined individual sgRNA p values from DESeq2 using Fisher's method followed by multiple hypothesis testing correction using the Benjamini-Hochberg procedure. The top resistance-associated genes were identified by having a count number >100 and log_2_ (fold change) >3 for functional enrichment. The sgRNA sequences were amplified using NEBNext® High-Fidelity 2X PCR Master Mix and subjected to massive parallel amplicon sequencing using Novogene Technology (Beijing, China). The sgRNA read count and hit calling were analyzed by the MAGeCK v0.5.7 algorithm 18. Pearson correlation coefficients of the normalized sgRNA read counts from the hGeCKO v2 plasmid library were determined (**Figure 1B**). For each biological sample type, two independent infection replicates (R1 and R2) were used. The number of unique sgRNAs, boxplot of the sgRNA normalized read counts for the hGeCKO v2 plasmid pool and cumulative probability distribution of library sgRNAs in the plasmid were performed to evaluate the representation of the hGeCKO v2 library in various samples by the R package (**Figure 1C-E**). The top 600 sgRNAs in Lenvatinib treated cells, 400 sgRNAs in primary tumors and 200 sgRNAs in lung metastases compared to control cells were used for the identification of coexpressed core genes. The trend group of sgRNAs was defined as those sgRNAs that had a count number of lung metastases > primary tumor > cell Lenvatinib.

### Genome-wide CRISPR/Cas9 library screening of Lenvatinib treatment for HCC cells in vitro and in vivo

In this study, we performed genome-wide CRISPR/Cas9 knockout library screening to identify critical genes associated with Lenvatinib resistance in human HCC. As shown in **Figure S1**, the hGeCKOa library was successfully cloned into HepG2 cells which had relatively higher minimum lethal concentration for Lenvatinib (**Figure S2**), to generate a mutant cell pool. It is hypothesized that the knockout of Lenvatinib resistance suppressor genes will enhance the development of drug resistance, while knockout of Lenvatinib resistance driver genes will sensitize HCC cells to Lenvatinib-induced cell death. The mutant cells were treated with 80 μM Lenvatinib or DMSO for 14 days, followed by sgRNA sequencing for positive and negative screening (**Figure 1A**). In the *in vivo* models, cells with or without hGeCKOa lentiviral library infection were injected into the livers of mice (**Figure 1A and S3**), accompanied with Lenvatinib treatment. At 5 weeks post-transplantation, the livers and lungs with primary tumors and lung metastases were harvested (**Figure S4**). Two random primary tumor and metastatic tumors from the lungs of the hGeCKOa mice treated with Lenvatinib were used for sgRNA sequencing. To investigate the sgRNA library dynamics in different sample types (control cells, Lenvatinib treated cells, primary tumors post Lenvatinib treatment, and lung metastases post Lenvatinib treatment), we compared the overall distributions of sgRNAs from all the sequenced samples. Cell samples with or without Lenvatinib treatment and primary tumors were tightly clustered with each other (**Figure 1B**). Comparable amounts of sgRNAs were detected in all samples with a characteristic small diversity (**Figure 1C and D**). Lung metastasis sample R2 exhibited a slight heterogeneity from the others (**Figure 1B-D**). The respective cumulative distribution functions revealed that the global patterns of sgRNA distributions in different sample types were closed (Kolmogorov-Smirnov [KS] test) (**Figure 1E**).

### Public data processing

To determine DUPS4 expression in HCC, sample data were obtained from the Cancer Genome Atlas (TCGA, http//gdc.cancer.gov/) and Gene Expression Omnibus (GEO, http://www.ncbi.nlm.nih.gov/geo, GSE25097, GSE63898) for analysis. To evaluate the significance of DUPS4 after Sorafenib treatment, the GEO datasets (GSE109211 and GSE151412) were analyzed. Data were Log_2_ converted and analyzed by R and GraphPad Prism 8 software. The relative expression levels were then determined. The edge R package was based on negative binomial distributions, an empirical Bayes estimation, exact tests, generalized linear models and quasi-likelihood tests.

### Functional enrichment analysis

The Database for Annotation, Visualization and Integrated Discovery (DAVID), which uses a modified Fisher's exact test followed by Benjamini-Hochberg multiple hypothesis testing correction, was used to determine the Kyoto Encyclopedia of Genes and Genomes (KEGG) pathway. Gene set enrichment analysis (GSEA) was performed to identify gene sets and pathways using the data obtained from the TCGA database. GSEA was performed using the Broad Institute Website (http://software.broadinstitute.org/gsea/index.jsp). Protein-protein interactions were performed with STRING (https://string-db.org/). Each gene in the list was weighted by its log fold change in expression.

### Cell culture and transfection

Cell lines (LO2, LM3, HepG2 and Huh7) were purchased from the Institute of Biochemistry and Cell Biology, Chinese Academy of Sciences, Shanghai, China. They were cultured in DMEM supplemented with 10% fetal bovine serum (penicillin and streptomycin) and incubated at 37°C in a humid atmosphere of 5% CO_2_. The DUSP4 knockout and overexpression vectors, which were constructed by Shanghai Generay Biotechnology Co., Ltd., were mixed with the pPACKH1 packaging plasmid and transfected into HEK293T cells. Three days later, according to the SBI instructions, viral particles were collected by concentrating the virus precipitation solution derived from *Letinus edodes*. TUNDUX viral transducers were used to infect cells. Positive cells were identified by puromycin screening.

### Immunofluorescence staining analysis

HepG2 cells were seeded into 24 well plates at a density of 5 × 10^3^ cells/well. Cells were fixed in 4% formaldehyde for 15 min at 37 °C, washed using PBS, blocked using 5% bovine serum albumin (Sangon Biotech Co., Ltd., Shanghai, China) for 30 min at room temperature, and incubated with cleaved-Caspase-3 primary antibodies at 4 °C overnight. Cells were then incubated with goat anti-rabbit immunoglobulin G H&L antibodies (Alexa Fluor® 488, Abcam; 1:1000, ab150077, UK) at 4 °C for 2 h. Nuclear staining was performed with DAPI (Sigma Aldrich; Merck KGaA, GER) at room temperature for 2 min. Then, the cells were washed three times using PBS and observed using an inverted fluorescence microscope (Olympus Corporation, Tokyo, Japan).

### Animal experiments

For the orthotopic tumor implantation model, 50 μl of 3 x 10^6^ HepG2-NC or HepG2-KO library cells were randomly injected into the livers of male BALB/C nude mice aged 6 to 8 weeks in 3 groups. To establish a subcutaneous xenograft model, a total of 5 × 10^6^ HepG2 cells stably expressing DUSP4 knockout (KO-DUSP4) or negative control (KO-NC) plasmids were resuspended in 100 μl of saline containing 50% Matrigel (BD Biosciences). Cells were subcutaneously injected into the right flank regions of male BALB/C nude mice aged 6 to 8 weeks. Tumor volumes were measured and recorded every 3 days to establish tumor growth, which were calculated by the following equation: tumor volume = long diameter*short diameter*short diameter/2. 14 days after injection, mice were treated with Lenvatinib (30mg/kg/d), Selumetinib (9.75mg/kg/d) or olive oil daily for 4 weeks by gavage. Then, on the 48^th^ day, the mice were sacrificed for tumor harvesting. Animal experiments were performed under the guidance of the Institutional Animal Care and Use Committee (IACUC), and ethical approval was obtained from the Institutional Assessment Committee.

### Reagents and in vitro experiments

The DUSP4 (ab229090) and GAPDH (ab8245) antibodies were purchased from Abcam and used as recommended. The pERK (#4695), Cleaved-Caspase-3 (#9664) and pMEK (Thr286, #9127) antibodies were purchased from Cell signaling and used as recommended. Cell proliferation, colony formation, Transwell migration assays, flow cytometry analysis, immunohistochemistry staining and Western blotting were performed as previously described 19. Lenvatinib (NO.417716-92-8) and the MEK inhibitor (Selumetinib, no.606143-52-6) were purchased from Selleckchem.

### Establishment of Lenvatinib Resistant HCC (LR-HCC) Cells

First, the half-maximal inhibitory concentration (IC50) of HepG2 and Huh7 cell lines to Lenvatinib were detected (**Figure S2C**). HepG2 or Huh7 cells were seeded into 96‐well plates and treated with various doses of Lenvatinib. After incubation for 72 hours, the cell viability was determined by CCK‐8. Then, HepG2 or Huh7 cells (1 × 10^4^ per well) were seeded into 6‐well palates and incubated with Lenvatinib concentrations just below their IC50. During the following weeks, the dosages of Lenvatinib were slowly increased. Over 4 months, HepG2 and Huh7 cell lines resistant to Lenvatinib (HepG2 LR and Huh7 LR) were established (**Figure S2C**). After establishment, these resistant cell lines were continuously cultured with the presence of Lenvatinib.

### Study participants and tissue samples

Tissue samples were obtained from HCC patients at Guangdong Provincial People's Hospital, the First Affiliated Hospital of Sun Yat-sen University and the Affiliated Cancer Hospital & Institute of Guangzhou Medical University. Resected liver tissues were obtained between 2013 and 2015. Patients administered with Lenvatinib therapy were from 2018 to 2020. All patients were not subjected to preoperative radiotherapy or preoperative chemotherapy. The tumor tissue samples were immediately preserved in liquid nitrogen and stored at -80 °C after biopsy or resection. Patient follow up was performed until June, 2021. Overall survival (OS) was defined as the period between hepatectomy and death while disease-free survival (DFS) was defined as the period between hepatectomy and the existence of tumor recurrence, detection of metastasis or cancer related death. This study was approved by the Institutional Review Board for the Protection of Human Subjects of Guangdong Provincial People's Hospital. We adhered to the tenets of the Declaration of Helsinki. Informed consent was obtained from the patients or their family members who agreed to the use of their samples in this study.

### Statistical analysis

The significance of continuous parameters presented as the mean ± SD *in vivo* and *in vitro* was determined by Student's t-test. The cell apoptosis rate detected by flow cytometry analysis was also compared by Student's t-test. The Fisher's exact test was used for categorical parameters. Survival curves were assessed by Kaplan-Meier analysis and compared by the log-rank test. P<0.05 was considered significant. Data analysis was performed using SPSS 22.0 software (IBM, USA). All experiments were independently repeated at least three times. In the figures, the symbols *, ** and *** represent p<0.05, p<0.01 and p< 0.001, respectively.

## Results

### Identification of key biological processes and genes associated with Lenvatinib resistance in HCC cells

**Figure 2:**
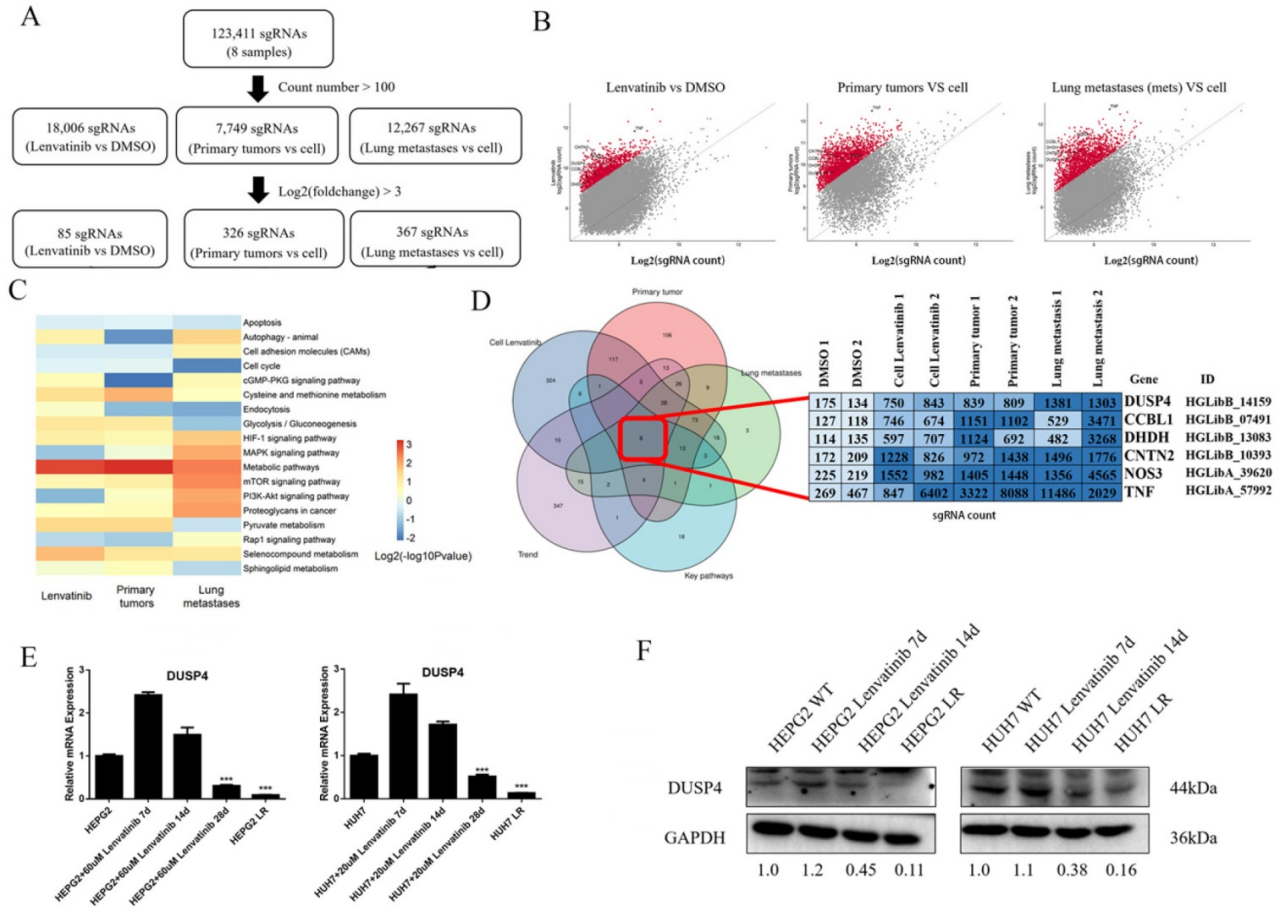
** Identification of key biological processes and genes associated with Lenvatinib resistance. (A)** Workflow of top enriched sgRNA selection. **(B)** Volcano plot of enriched sgRNAs identified in Lenvatinib resistant HCC cells, primary tumors and lung metastasis. **(C)** Compared to the control HCC cells, the differentially expressed sgRNAs were enriched in 18 pathways. **(D)** Identification of key genes related to Lenvatinib resistance among Lenvatinib treated cells, primary tumors post Lenvatinib treatment, lung metastases post Lenvatinib treatment, key pathways and developing trend genes.** (E and F)** Verification of DUSP4 expression at mRNA and protein levels in Lenvatinib-treated (60μM in HEPG2 and 20 μM in HUH7) HCC cells and resistant cells. *** represents p< 0.001.

All 123,411 sgRNAs were detected by sgRNA sequencing of the 8 samples (**Figure 2A**). With a cutoff value that counted numbers > 100, a total of 18,006 sgRNAs were detected in Lenvatinib vs Control cell group, while 7,749 sgRNAs in Primary tumor vs Control cell group and 12,267 sgRNAs in Lung metastases vs Control cell group. We identified 85, 326 and 367 sgRNA constructs from the three groups, respectively, with a log_2_ (fold change) > 3 for enrichment of KEGG pathways (**Figure 2B and C**). Compared to the control HCC cells, the differentially expressed sgRNAs were enriched in 18 pathways, including apoptosis, autophagy, cell adhesion molecules, cell cycle, cGMP-PKG signaling pathway, cysteine and methionine metabolism, endocytosis, glycolysis/gluconeogenesis, HIF-1 signaling pathway, mitogen-activated protein kinases (MAPK) signaling pathway, metabolic pathways, mTOR signaling pathway, phosphatidylinositol 3-kinase (PI3K)-protein kinase B (Akt) signaling pathway, proteoglycans in cancer, pyruvate metabolism, Rap1 signaling pathway, selenocompound metabolism, and sphingolipid metabolism (**Figure 2C**). Six sgRNAs were identified as coexpressed core genes, including sgRNAs of DUSP4, CCBL1, DHDH, CNTN2, NOS3 and TNF (**Figure 2D**). Moreover, DUSP4 was found to be significantly decreased at the mRNA and protein levels in HCC cells continuously treated with Lenvatinib, especially in resistant cells comparing to control cells (**Figure 2E and F**), indicating that loss of DUSP4 might induce Lenvatinib resistance in HCC.

### DUSP4 deficiency enhanced Lenvatinib resistance in HCC cells

**Figure 3:**
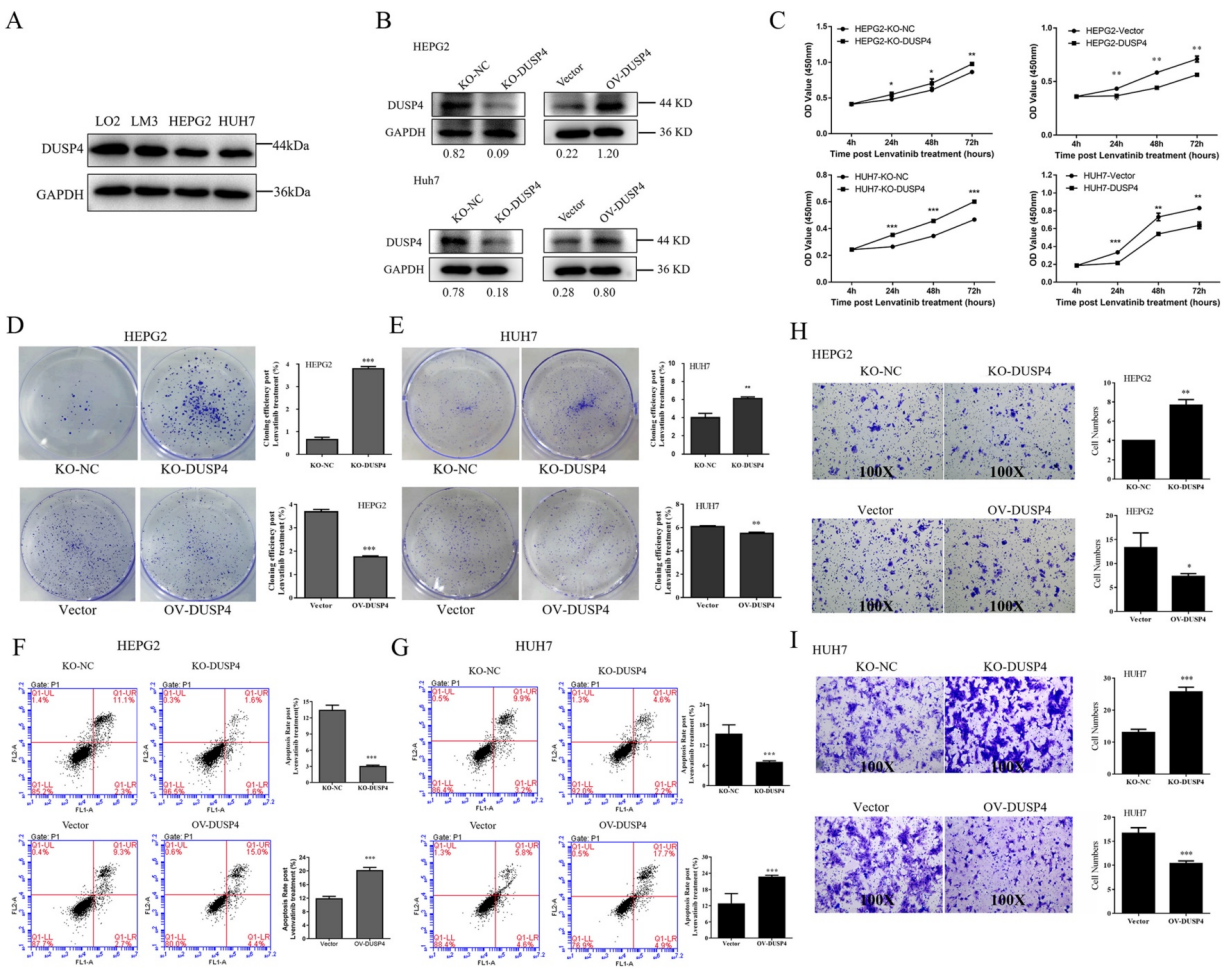
** DUSP4 deficiency enhanced Lenvatinib resistance. (A)** HepG2 cells and Huh7 cells had expressed medium level of DUSP4.** (B)** Construction of DUSP4 knockout and overexpression in HepG2 cells and Huh7 cells. **(C-E)** DUSP4 deficiency in HCC cells enhanced Lenvatinib resistance and helped maintain cell proliferation, survival and migration ability, as indicated by CCK8 assay, colony formation assay, flow cytometry analysis and the Transwell assay. All cells were continuously treated with Lenvatinib (20 µM) 24 hours before experiments. *, ** and *** represent p<0.05, p<0.01 and p< 0.001.

To validate the role of DUSP4 in Lenvatinib resistance, we constructed DUSP4 knockout and overexpression in HCC cells that had medium level of DUSP4 expression (HepG2 cells and Huh7 cells) (**Figure 3A and B**). The CCK8 assay revealed that DUSP4 knockout HCC cells had a higher proliferative capacity during the treatment of Lenvatinib, while DUSP4 overexpression HCC cells showed impaired proliferation under Lenvatinib treatment, compared to the control cells (**Figure 3C**). The colony formation assay also showed similar results, indicating that the loss of DUSP4 restored cell growth and proliferation capacity during the challenge of Lenvatinib (**Figure 3D and E**). Furthermore, DUSP4 deficiency inhibited Lenvatinib-induced apoptosis, whereas the overexpression of DUSP4 sensitized HCC cells to Lenvatinib-induced apoptosis (**Figure 3F and G**). The sgRNA sequencing and analysis revealed that DUSP4 was also associated with lung metastases. We then assessed its role in cell migration during Lenvatinib using the Transwell assay (**Figure 3H and I**). DUSP4 knockout HCC cells showed an enhanced migration ability compared to KO-NC cells under Lenvatinib treatment, while overexpression of DUSP4 sensitized HCC cells to Lenvatinib-induced inhibition of migration. These results implied that DUSP4 deficiency in HCC cells enhanced Lenvatinib resistance and restored cell proliferation, survival and migration capacity.

### Inhibition of DUSP4 impairs the in vivo anti-tumor effects of Lenvatinib

**Figure 4:**
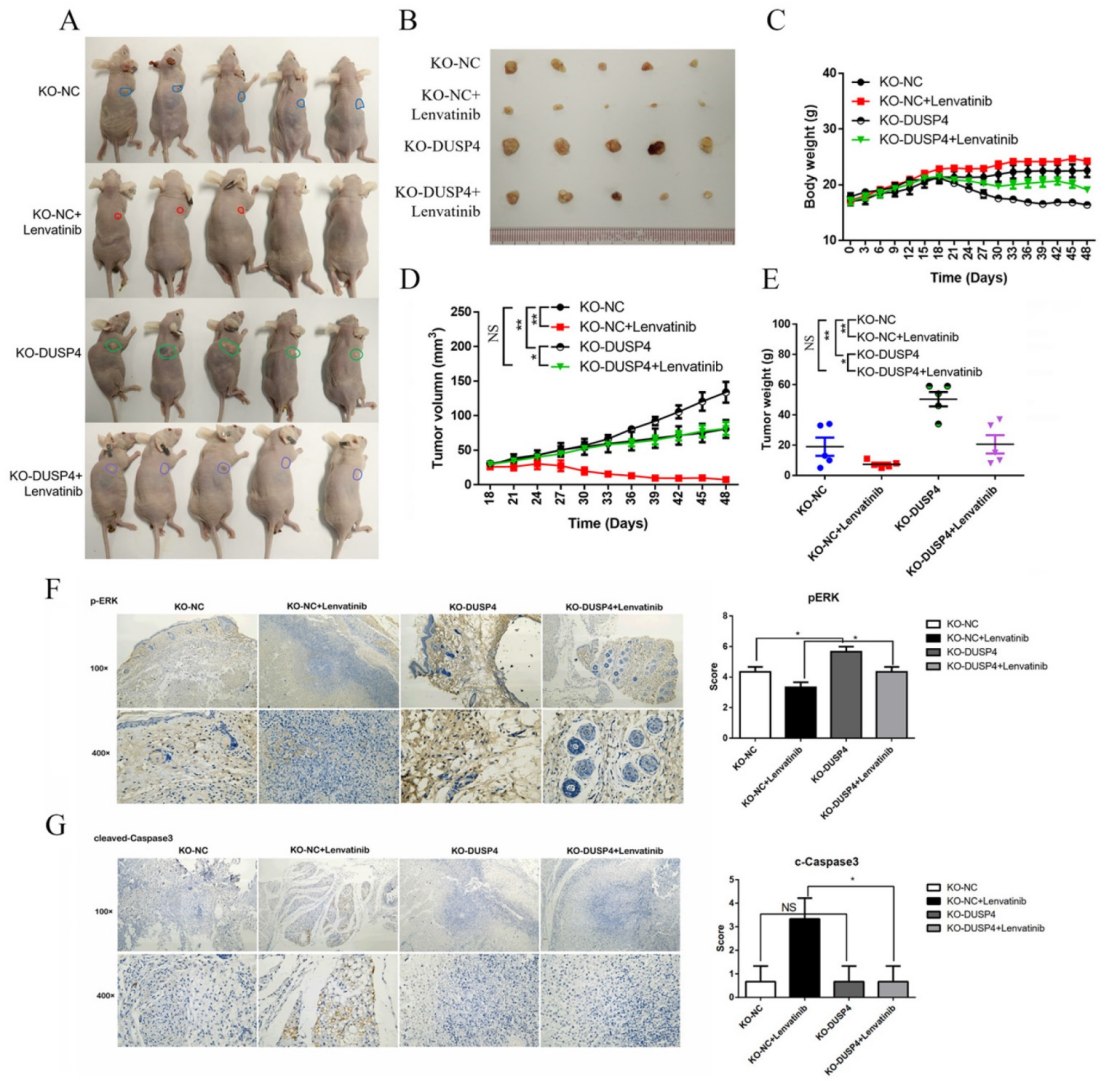
** Inhibition of DUSP4 impairs the antitumor effects of Lenvatinib *in vivo*. (A and B)** Lenvatinib treatment reduced tumor masses, which was abrogated by DUSP4 knockout. **(C)** KO-DUSP4 mice showed a prominent loss of body weight. Tumor volumes **(D)** and tumor weights **(E)** of mice in each group. **(F and G)** Immunohistochemical staining analysis showed that KO-DUSP increased p-ERK1/2 and decreased cleaved Caspase-3 in tumor tissue, even after the treatment of Lenvatinib. * and NS represent p<0.05 and p>0.05.

To evaluate the *in vivo* effects of DUSP4 deficiency on Lenvatinib resistance, HCC cells (NC-sgRNA and DUSP4-sgRNA) were subcutaneously inoculated into nude mice in four groups. When the tumors were palpated, the mice were treated with the Lenvatinib (30 mg/kg) and olive oil (negative control) by gavage. Lenvatinib treatment significantly reduced the tumor mass in KO-NC Lenvatinib group compared to KO-NC group, while the reduced tumor mass could be abrogated in KO-DUSP4 Lenvatinib group compared to the KO-NC Lenvatinib group (**Figure 4A and B**). KO-DUSP4 group showed a prominent loss of body weight (**Figure 4C**). Additionally, The KO-DUSP4 group also showed enhanced tumor growth compared to the KO-NC group, implying that DUSP4 might be a tumor suppressor (**Figure 4D and E**). Furthermore, immunohistochemical staining analysis showed that KO-DUSP4 group increased p-ERK1/2 and decreased cleaved Caspase-3 levels in tumor tissues even with Lenvatinib treatment (**Figure 4F and G**). These results implied that DUSP4 may act as a tumor suppressor, and the loss of DUSP4 could induce Lenvatinib resistance and enhance tumor growth *in vivo*.

### DUSP4 depletion enhanced Lenvatinib resistance by activating the MAPK/ERK pathway

**Figure 5:**
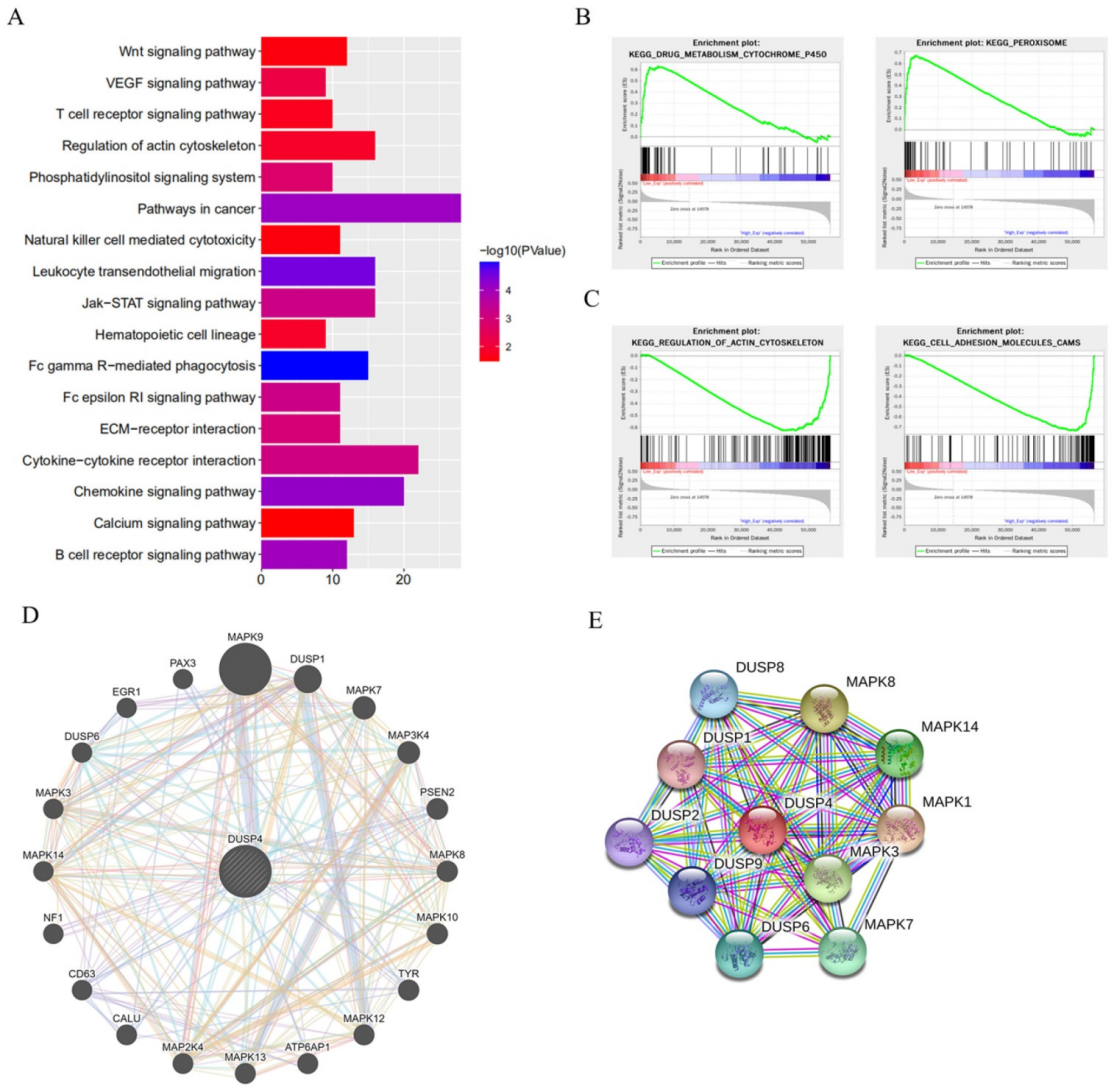
** DUSP4 depletion enhanced Lenvatinib resistance by activating the MAPK/ERK pathway. (A)** The KEGG pathway analysis showed that DUSP4 participated in Pathways in cancer, Cytokine-cytokine receptor interaction, Regulation of actin cytoskeleton and other pathways related cancer progression. **(B-C)** The GSEA enrichments demonstrated that DUSP4 is enriched in drug metabolism, Peroxisome, Regulation of actin cytoskeleton and Cell adhesion molecules. **(D)** The GeneMANIA network showed that DUSP4 is closely associated with MAPK family.** (E)** protein-protein interaction network revealed that DUSP4 is closely related to DUSP family and MAPK family.

To elucidate the mechanism associated with DUSP4 deficiency induced Lenvatinib resistance, KEGG pathway, GSEA enrichment analysis, GeneMANIA network and protein-protein interaction network analyses were performed (**Figure 5**). DUSP4 was found to be involved in cytokine-cytokine receptor interaction, regulation of actin cytoskeleton and other pathways associated with cancer progression (**Figure 5A**) by KEGG pathway analysis, while the GSEA enrichment analysis showed that DUSP4 was enriched in drug metabolism, peroxisome, regulation of actin cytoskeleton and cell adhesion molecules (**Figure 5B and C**). In particular, GeneMANIA network and protein-protein interaction network showed that DUSP4 was closely associated with the MAPK family (**Figure 5D and E**) and that DUSP4 was reported to be a MAP kinase phosphatase. As shown in **Figures S5 and S6**, the GO terms and KEGG pathways enriched by differentially expressed sgRNAs revealed that Lenvatinib resistant (LR) cells and LR related metastasis could be associated with the MAPK signaling pathway and various processes. According to previous study of DUSP4 20-22, we focused on DUSP4's role in MEK/ERK pathway.

### Selumetinib, a MEK1/2 inhibitor, reversed DUSP4 loss associated Lenvatinib resistance in vitro and in vivo

**Figure 6:**
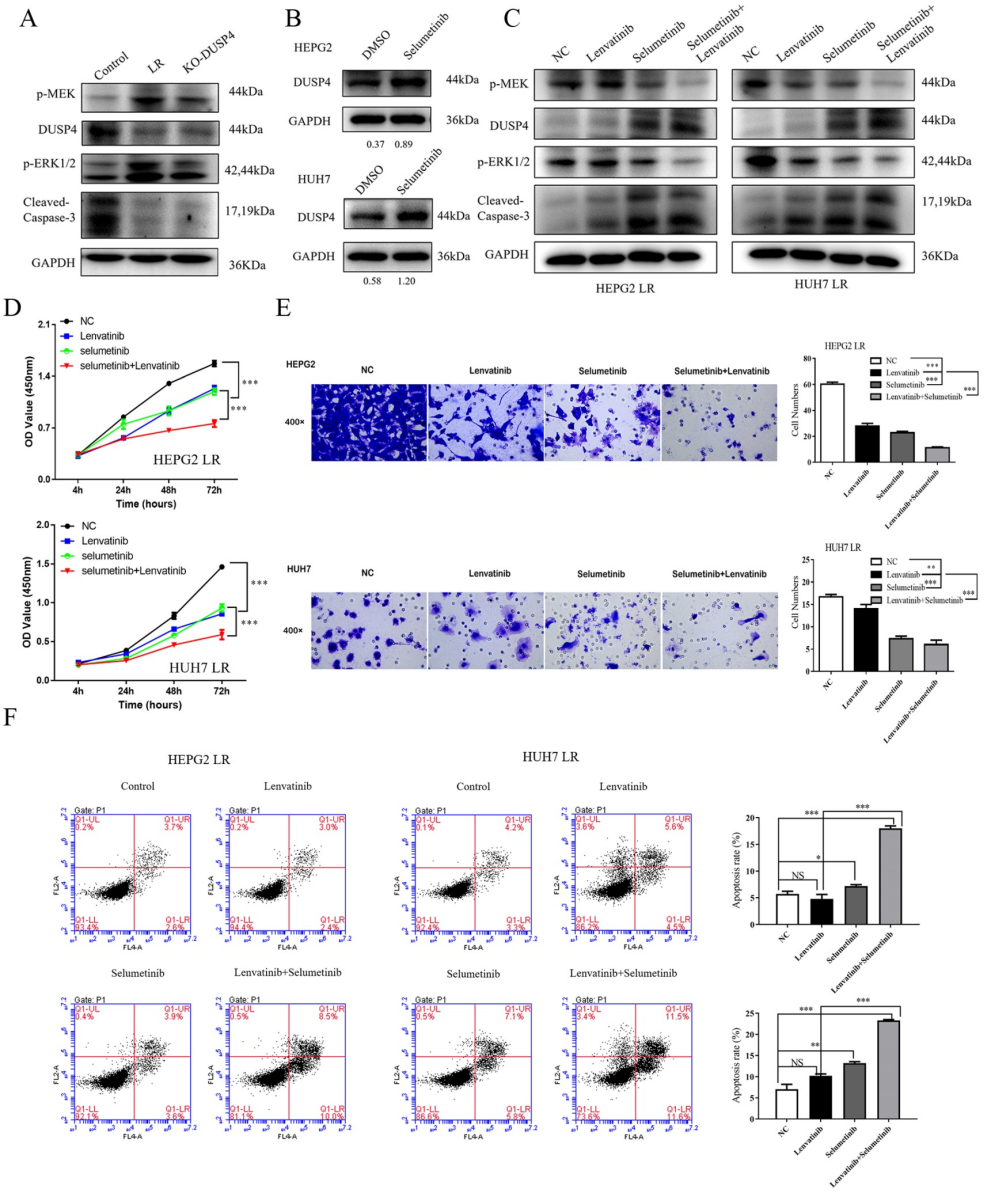
** Selumetinib reversed Lenvatinib resistance associated with DUSP4 loss. (A)** Lenvatinib-resistant (LR) HCC and KO-DUSP4 cells showed upregulation of p-MEK and pERK1/2. **(B)** DUSP4 was upregulated during Selumetinib treatment. Western blotting showed that Selumetinib could reverse activation of the MAPK/ERK pathway associated with DUSP4 loss. **(C)** Expression of DUSP4 and cleaved caspase-3 was upregulated, while p-MEK and pERK1/2 were downregulated during Selumetinib treatment in LR cells. **(D-F)** Selumetinib reversed cell proliferation, apoptosis resistance and migration associated with DUSP4 deficiency, as indicated by the CCK8 assay, Transwell assay and flow cytometry analysis. LR, Lenvatinib-resistant. Cells were continuously treated with Lenvatinib (20 µM) and Selumetinib (2 µM) in designated group. Student's t-test **P < 0.01, ***p< 0.001. NS represents p>0.05.

**Figure 7:**
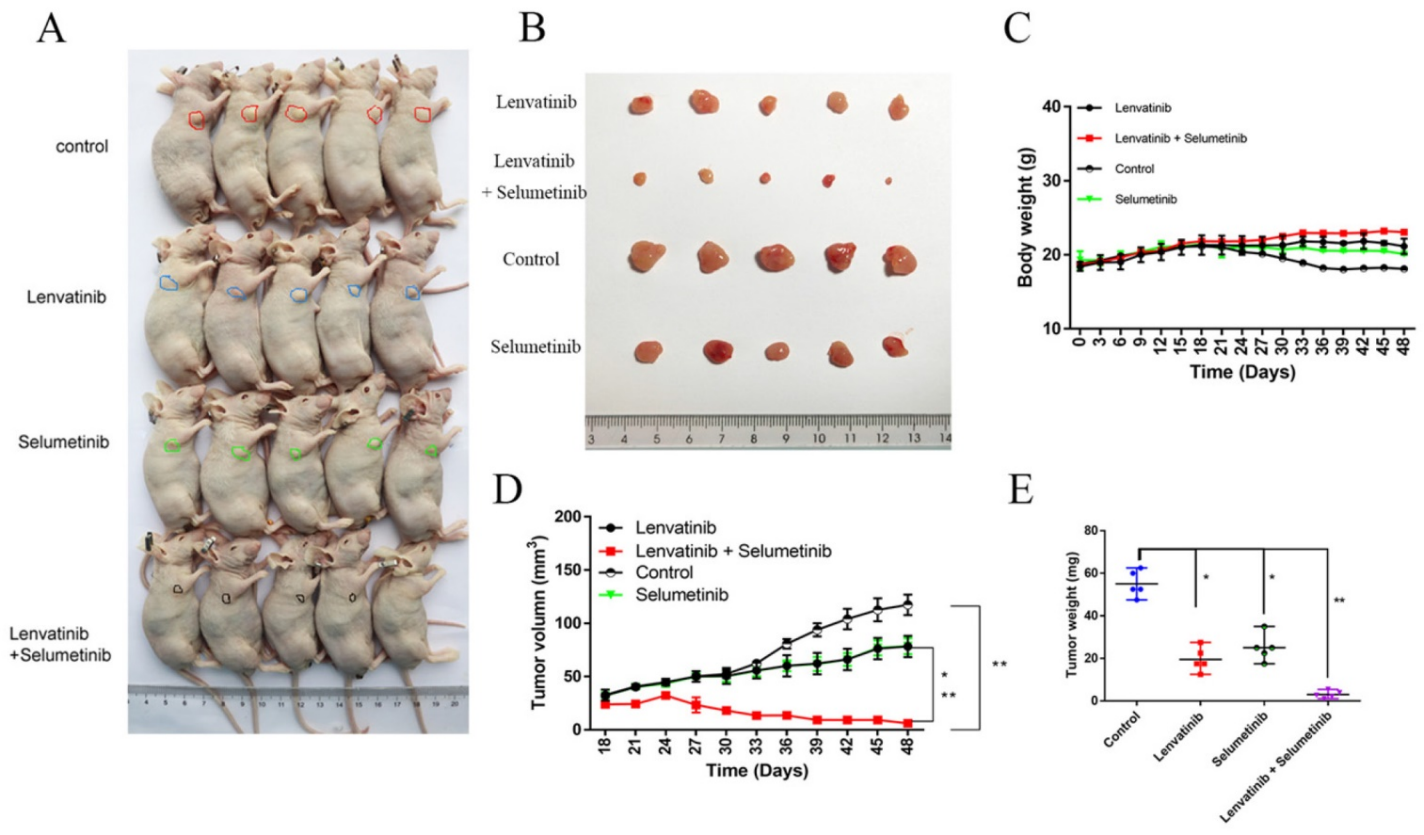
** Selumetinib, a MEK1/2 inhibitor, reversed DUSP4 loss associated Lenvatinib resistance* in vivo*. (A and B)** Both Lenvatinib and Selumetinib could slightly inhibited KO-DUSP4 HCC tumorigenicity. Lenvatinib and Selumetinib combination therapy effectively abolished HCC growth in the nude mice model. **(C)** The body weights of mice in the three drug-treated groups remained unchanged. Lenvatinib and Selumetinib combination therapy obviously reduced tumor volumes **(D)** and tumor weights **(E)**. Student's t-test *P < 0.05, **P < 0.01.

As shown in **Figure 6A**, p-MEK and pERK1/2 were upregulated in both LR HCC and KO-DUSP4 cells. To further determine whether Lenvatinib resistance induced by DUSP4 deficiency was dependent on the activation of MAPK/ERK pathway, we used a MEK1/2 inhibitor, Selumetinib, to block the MAPK/ERK pathway in LR HCC cells. DUSP4 upregulation was observed when comparing HCC cells and HCC cells treated with 2 μM Selumetinib, indicating that MEK inhibition could upregulate DUSP4 expression in HCC cells (**Figure 6B**). Along with Selumetinib treatment, DUSP4 and cleaved-Caspase 3 expression were upregulated while p-MEK and pERK1/2 were downregulated, indicating a reversion of Lenvatinib resistance (**Figure 6C**). The CCK8 assay showed that LR HCC cells were only partly resistant to Lenvatinib treatment as evidenced by the proliferating cells at a medium level, while treating with both Selumetinib and Lenvatinib showed significant inhibition of cell proliferation (**Figure 6D**). The combination treatment also significantly sensitized LR HCC cells to Selumetinib-inhibited cell migration (**Figure 6E**) and induced apoptosis (**Figure 6F**). These findings indicated that the decreased apoptosis and restored cell proliferation induced by DUSP4 deficiency was dependent on the activation of MAPK/ERK pathway. Taken together, these findings suggested that DUSP4 deficiency induced Lenvatinib resistance through the activation of MAPK/ERK pathway and inhibition of MEK helped overcome Lenvatinib resistance in treating HCC. Next, we performed subcutaneous injection of HEPG2 KO-DUSP4 cells in nude mice. When tumors were palpable, mice were divided into four groups randomly and were treated with Lenvatinib alone, Selumetinib alone, Lenvatinib and Selumetinib, and empty controls. Lenvatinib and Selumetinib alone could reduce HCC growth in vivo while combined treatment of Lenvatinib and Selumetinib fundamentally halted HCC growth in vivo (**Figure 7A-E**). These data together confirmed that Selumetinib could reversed DUSP4 loss associated Lenvatinib resistance.

### DUSP4 loss was associated with clinicopathologic characteristics, patient prognosis and response to Lenvatinib

**Figure 8:**
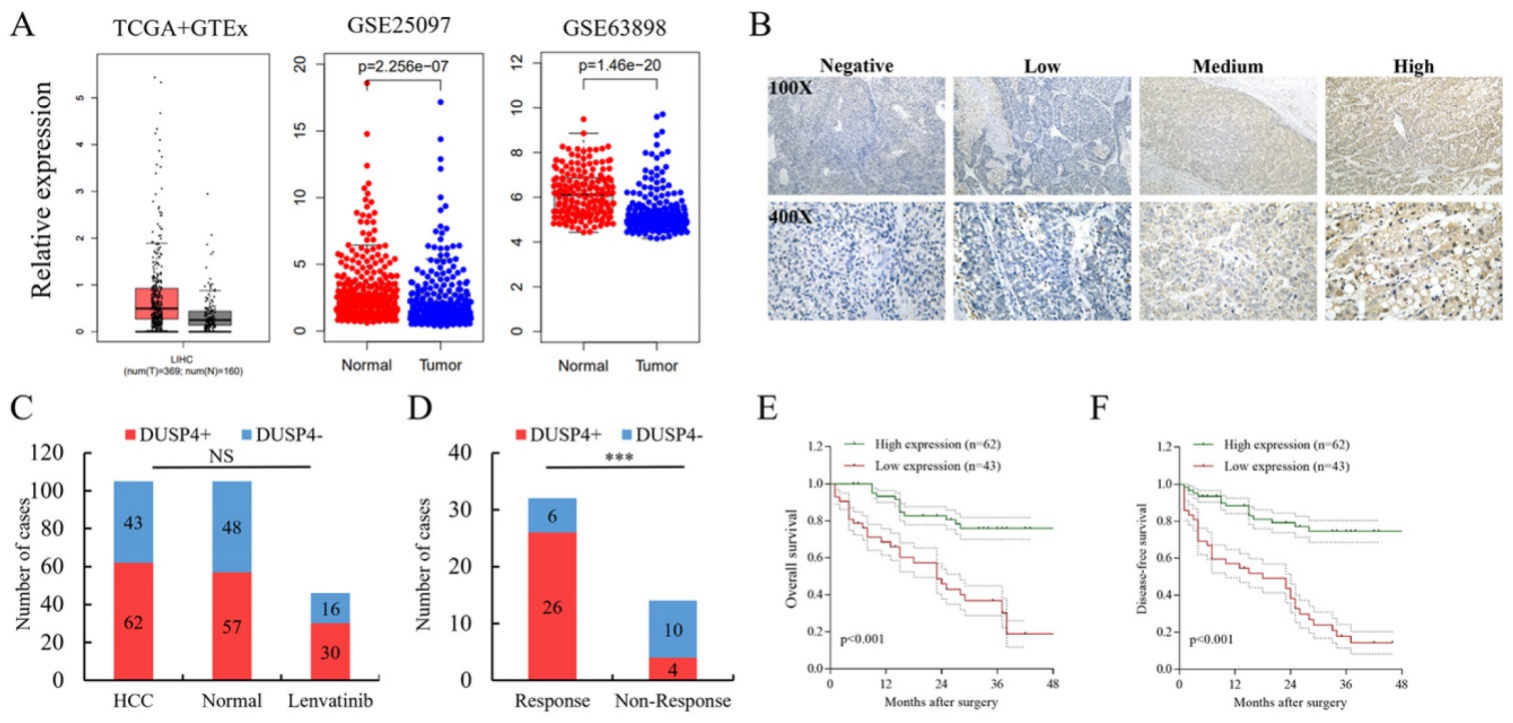
** DUSP4 loss is associated with response to Lenvatinib and prognosis.** (**A**) The expression of DUSP4 was downregulated in HCC tissues. **(B)** Representative images of DUSP4 staining in HCC specimens. **(C)** There was no significant difference in DUSP4 expression among HCC tissues, normal liver tissues or biopsies of HCC patients before lenvatinib treatment. **(D)** Responders to lenvatinib treatment showed higher DUSP4 expression levels than nonresponders.** (E and F)** Low expression levels of DUSP4 were associated with poor OS and DFS. *** represents p< 0.001.

Based on the databases of TCGA and GEO, the expression of DUSP4 was downregulated (GSE25097, GSE63898) in HCC tissues (**Figure 8A**). The validation of clinical samples showed that there is no significant difference in DUSP4 expression among HCC tissues, normal liver tissues or biopsies of HCC patients with Lenvatinib treatment (**Figure 8B and C**). However, patients who responded to Lenvatinib exhibited higher DUSP4 expression levels than those without response (**Figure 8D**). Furthermore, low expression levels of DUSP4 in HCC was associated with inferior OS and DFS in HCC patients following surgical resection (**Figure 8E and F**). We also investigated whether DUSP4 deficiency was associated with sorafenib resistance by analyzing the expression of DUSP4 in a cohort of HCC patients received sorafenib treatment. The data from GSE109211 showed that DUSP4 expression in HCC tissues was comparable between the sorafenib group and the placebo group, while HCC patients that were non-responsive to sorafenib had lower DUSP4 expression levels than responders (**Figure S7A**). In addition, high-DUSP4 HCC patients in the course of Sorafenib treatment had a better OS with a very slight trend toward significance (p=0.59) than low-DUSP4 HCC patients (**Figure S7B**). Consistently, Hep3B cells that were resistant to Sorafenib had lower levels of DUSP4 as indicated by GSE1512 data (**Figure S7C**). The pubic data shown in **Figure S7** revealed that DUSP4 could be a marker for Sorafenib sensitivity. These findings indicated that DUSP4 deficiency correlated with higher possibility of Lenvatinib and sorafenib resistance, which may act as a biomarker in predicting drug resistance in HCC therapy.

## Discussion

**Figure 9:**
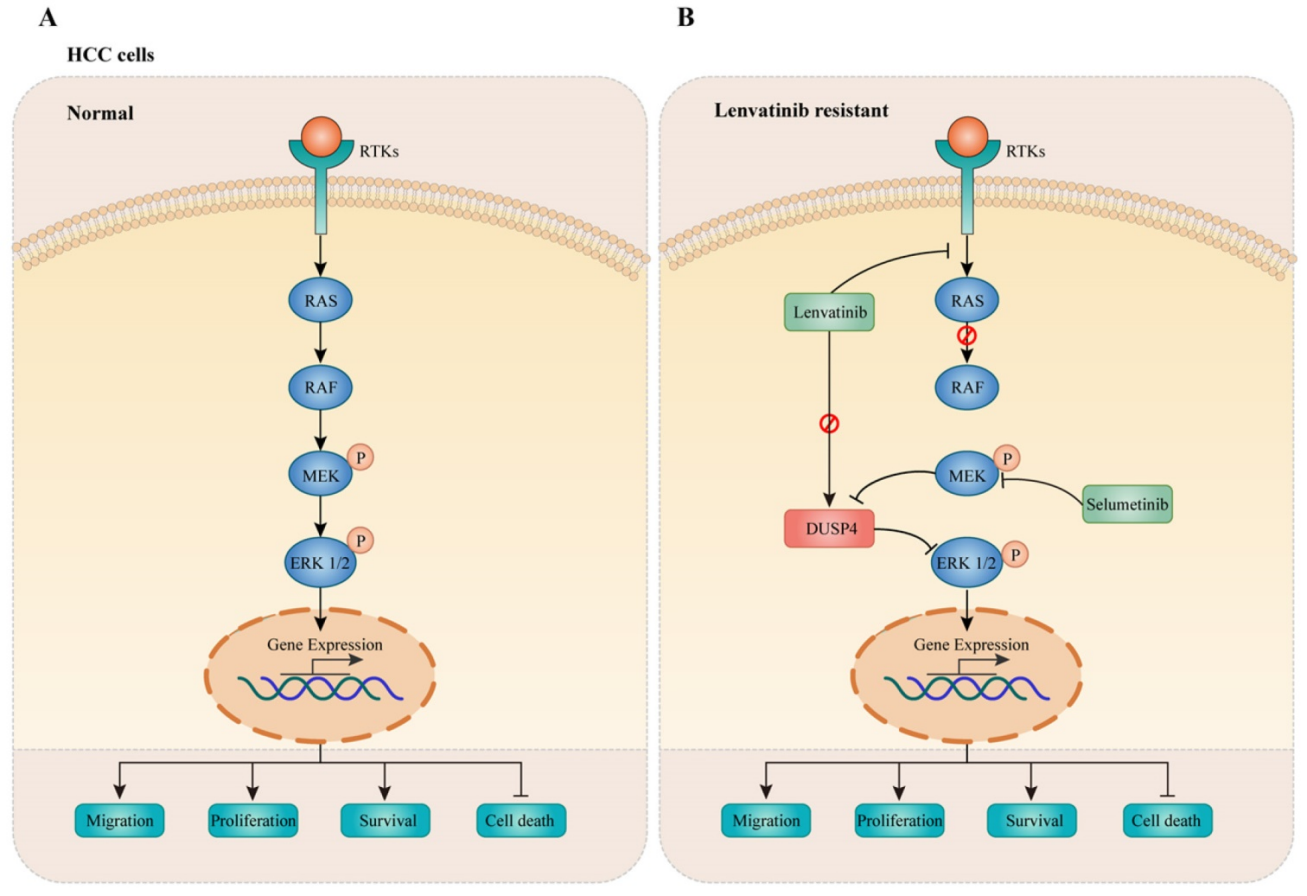
** DUSP4 deficiency results in ERK activation leading to Lenvatinib resistance. (A)** In normal HCC cells, receptor tyrosine kinases (RTKs) activate RAS-RAF to enhance phosphorylation of MEK and ERK1/2, leading to cancer progression. **(B)** Lenvatinib resistance was accompanied with DUSP4 downregulation, accounting for the activation of the MAPK/ERK pathway, which resulted in enhanced cell migration, proliferation, survival and inhibited cell death. The inhibition of MEK by Selumetinib enhanced treatment sensitivity to Lenvatinib in DUSP4 deficiency HCC.

In this study, a genome-wide CRISPR/Cas9 knockout screening system was used to identify critical genes associated with Lenvatinib resistance in HCC. Importantly, DUSP4 deficiency was observed in Lenvatinib resistant HCC cells both *in vitro* and *in vivo*, leading to cell survival, migration and prevention of apoptosis. Mechanistically, DUSP4 deficiency activated the MAPK/ERK pathway, while inhibition of ERK enhanced treatment sensitivity to Lenvatinib in KO-DUSP4 HCC cells. These findings showed that the effect of DUSP4 deficiency on Lenvatinib resistance was dependent on MEK and ERK activity (**Figure 9**). Therefore, DUSP4 deficiency is a major regulatory mechanism for Lenvatinib resistance in HCC.

DUSP4 is a member of the dual specificity protein phosphatase subfamily that is involved in the inactivation of the corresponding target kinases, including the MAPK cascade 23. Dual specificity protein phosphatase subfamily showed various functions. Studies have documented that low DUSP4 expression levels are found in more aggressive cancers while the knockdown of DUSP4 promotes tumor development and progression in colorectal cancer and glioblastoma, suggesting the role of DUSP4 as a tumor suppressor 24-25. In contrast, Chen et al. have reported that miR-1226-3p promotes sorafenib sensitivity of HCC through downregulation of DUSP4 expression 26. In this study, we found that DUSP4 knockout enhanced tumor growth in HCC xenograft nude mouse models without Lenvatinib treatment. In addition, the inhibition of DUSP4 suppressed the anti-tumor effect of Lenvatinib, indicating that DUSP4 deficiency is a critical driver for treatment failure. Similarly, Balko et al. demonstrated that DUSP4 down-regulation is correlated with worse patient responses to neoadjuvant chemotherapy for breast cancer 22. Based on these findings, a complete functional DUSP4 is necessary for successful treatment efficacy of chemotherapy or targeted therapy. In our study, DUSP4 play as HCC tumor suppressor, while its deficiency also greatly cut down treating effect of Lenvatinib. Moreover, DUSP4 might be a potential crucial biomarker for predicting patient responses to target therapy. Further studies should be conducted to elucidate the molecular mechanisms by which Lenvatinib treatment suppresses DUSP4 expression.

The MAPK pathway consists of a cascade of protein kinases including RAS, RAF, mitogen-activated protein/extracellular signal-regulated kinase (MEK) and the extracellular signal-regulated kinase (ERK). The MAPK pathway plays an important role in regulating cell proliferation, differentiation and apoptosis. Dysregulation of the MAPK pathway may lead to tumorigenesis by phosphorylating a spectrum of substrates, mostly oncogenic transcription factors 27. Blocking the MAPK pathway is a potential clinical strategy for treating cancer. Some MAPK inhibitors, such as BRAF inhibitors are being evaluated in clinical trials 28. A previous study revealed that Lenvatinib resistance of HCC was mediated by the upregulation of VEGFR2 expression and its downstream pathway (RAS/MEK/ERK) 28. In this study, we found that the phosphorylation level of ERK, the central factor in the MAPK pathway, was elevated by DUSP4 suppression in resistant HCC cells. Inhibition of ERK phosphorylation successfully eliminated the drug resistance induced by DUSP4 reduction. Therefore, blocking the upstream signal of the MAPK pathway is not sufficient for eradicating cancer cells, as a complementary mechanism will reactivate the downstream signal of MAPK pathway. Combination therapy using Lenvatinib and MEK inhibitors may be a promising therapeutic strategy to overcome Lenvatinib resistance.

We also screened for other critical signaling pathways and genes associated with Lenvatinib resistance in HCC, such as HIF-1 signaling and NOS3. Consistent with our results, it has been reported that HIF-1 promotes the enrichment of cancer stem cells that are more resistant to cytotoxic chemotherapy 29. NOS3 has also been shown to be involved in chemoresistance in cancer 30. It is important to integrate these drug resistant related genes and establish a predictive model for evaluating clinical responses to targeted therapy.

Studies have shown that various genes and pathways are involved in HCC with Lenvatinib resistance, all of which could revealed the mechanism from different ways 31,32. In conclusion, our study has identified DUSP4 as the vital gene associated with Lenvatinib resistance in HCC by employing the genome-wide CRISPR/Cas9 library screening, which may provide an important reference for overcoming tyrosine kinase inhibitors resistance. DUSP4 deficiency mediated Lenvatinib resistance by re-activating ERK and MEK in HCC with Lenvatinib treatment. Further clinical studies of combination therapy with Lenvatinib and MEK inhibitors for HCC may be crucial to find an effective treatment to overcome drug resistance.

## Supplementary Material

Supplementary figures.

## Abbreviations

HCC: hepatocellular carcinoma
RNAi: RNA interference
CRISPR/Cas9: CRISPR-associated nuclease Cas9
OS: Overall survival
DFS: disease-free survival
PCR: polymerase chain reaction
sgRNA: small guide RNA
KEGG: Kyoto Encyclopedia of Genes and Genomes
GSEA: Gene Set Enrichment Analysis
TCGA: The Cancer Genome Atlas
GEO: Gene Expression Omnibus
MAPK: mitogen-activated protein kinases
MEK: mitogen-activated protein/extracellular signal-regulated kinase
ERK: extracellular signal-regulated kinase
LR: Lenvatinib resistant

## References

[1] Yang JD, Hainaut P, Gores GJ (2019). A global view of hepatocellular carcinoma: trends, risk, prevention and management. Nat Rev Gastroenterol Hepatol.

[2] Bouattour M, Mehta N, He AR (2019). Systemic Treatment for Advanced Hepatocellular Carcinoma. Liver Cancer.

[3] Josep M Llovet, Robin Kate Kelley, Augusto Villanueva (2021). Hepatocellular Carcinoma. Nat Rev Dis Primers.

[4] Bruix J, Qin S, Merle P (2017). Regorafenib for patients with hepatocellular carcinoma who progressed on sorafenib treatment (RESORCE): a randomised, double-blind, placebo-controlled, phase 3 trial. Lancet.

[5] Zhao Y, Zhang YN, Wang KT (2020). Lenvatinib for hepatocellular carcinoma: From preclinical mechanisms to anti-cancer therapy. Biochim Biophys Acta Rev Cancer.

[6] Matsui J, Yamamoto Y, Funahashi Y (2008). E7080, a novel inhibitor that targets multiple kinases, has potent antitumor activities against stem cell factor producing human small cell lung cancer H146, based on angiogenesis inhibition. Int J Cancer.

[7] Tohyama O, Matsui J, Kodama K (2014). Antitumor activity of lenvatinib (e7080): an angiogenesis inhibitor that targets multiple receptor tyrosine kinases in preclinical human thyroid cancer models. J Thyroid Res.

[8] Al-Salama ZT, Syed YY, Scott LJ (2019). Lenvatinib: A Review in Hepatocellular Carcinoma. Drugs.

[9] Motzer RJ, Hutson TE, Glen H (2015). Lenvatinib, everolimus, and the combination in patients with metastatic renal cell carcinoma: a randomised, phase 2, open-label, multicentre trial. Lancet Oncol.

[10] Kudo M, Finn RS, Qin S (2018). Lenvatinib versus sorafenib in first-line treatment of patients with unresectable hepatocellular carcinoma: a randomised phase 3 non-inferiority trial. The Lancet.

[11] Khan HY, Ge J, Nagasaka M (2019). Targeting XPO1 and PAK4 in 8505C Anaplastic Thyroid Cancer Cells: Putative Implications for Overcoming Lenvatinib Therapy Resistance. Int J Mol Sci.

[12] Chen S, Sanjana NE, Zheng K (2015). Genome-wide CRISPR Screen in a Mouse Model of Tumor Growth and Metastasis. Cell.

[13] Wang T, Wei JJ, Sabatini DM (2014). Genetic screens in human cells using the CRISPR-Cas9 system. Science.

[14] Zhao Z, Zhang D, Wu F (2021). Sophoridine suppresses lenvatinib-resistant hepatocellular carcinoma growth by inhibiting RAS/MEK/ERK axis via decreasing VEGFR2 expression. J Cell Mol Med.

[15] Wei L, Lee D, Law C (2019). Genome-wide CRISPR/Cas9 library screening identified PHGDH as a critical driver for Sorafenib resistance in HCC. Nature Communications.

[16] Sanjana NE, Shalem O, Zhang F (2014). Improved vectors and genome-wide libraries for CRISPR screening. Nat Methods.

[17] Goodspeed A, Jean A, Costello JC (2019). A Whole-genome CRISPR Screen Identifies a Role of MSH2 in Cisplatin-mediated Cell Death in Muscle-invasive Bladder Cancer. European Urology.

[18] Li W, Xu H, Xiao T (2014). MAGeCK enables robust identification of essential genes from genome-scale CRISPR/Cas9 knockout screens. Genome Biol.

[19] Huang S, Zhang C, Sun C (2020). Obg-like ATPase 1 (OLA1) overexpression predicts poor prognosis and promotes tumor progression by regulating P21/CDK2 in hepatocellular carcinoma. Aging (Albany, NY.).

[20] Gupta R, Bugide S, Wang B (2019). Loss of BOP1 confers resistance to BRAF kinase inhibitors in melanoma by activating MAP kinase pathway. Proceedings of the National Academy of Sciences.

[21] Balko JM, Cook RS, Vaught DB (2012). Profiling of residual breast cancers after neoadjuvant chemotherapy identifies DUSP4 deficiency as a mechanism of drug resistance. Nature Medicine.

[22] Balko JM, Schwarz LJ, Bhola NE (2013). Activation of MAPK Pathways due to DUSP4 Loss Promotes Cancer Stem Cell-like Phenotypes in Basal-like Breast Cancer. Cancer Research.

[23] Chen HF, Chuang HC, Tan TH (2019). Regulation of Dual-Specificity Phosphatase (DUSP) Ubiquitination and Protein Stability. Int J Mol Sci.

[24] Xue Z, Vis DJ, Bruna A (2018). MAP3K1 and MAP2K4 mutations are associated with sensitivity to MEK inhibitors in multiple cancer models. Cell Res.

[25] Waha A, Felsberg J, Hartmann W (2010). Epigenetic downregulation of mitogen-activated protein kinase phosphatase MKP-2 relieves its growth suppressive activity in glioma cells. Cancer Res.

[26] Chen X, Tan W, Li W (2019). miR-1226-3p Promotes Sorafenib Sensitivity of Hepatocellular Carcinoma via Downregulation of DUSP4 Expression. J Cancer.

[27] Braicu C, Buse M, Busuioc C (2019). Comprehensive Review on MAPK: A Promising Therapeutic Target in Cancer. Cancers (Basel).

[28] Yaeger R, Corcoran RB (2019). Targeting Alterations in the RAF-MEK Pathway. Cancer Discov.

[29] Markman JL, Rekechenetskiy A, Holler E (2013). Nanomedicine therapeutic approaches to overcome cancer drug resistance. Adv Drug Deliv Rev.

[30] Zheng Y, Dai Y, Liu W (2019). Astragaloside IV enhances taxol chemosensitivity of breast cancer via caveolin-1-targeting oxidant damage. J Cell Physiol.

[31] Jin H, Shi Y, Lv Y (2021). EGFR activation limits the response of liver cancer to lenvatinib. Nature.

[32] Lu Y, Shen H, Huang W (2021). Genome-scale CRISPR-Cas9 knockout screening in hepatocellular carcinoma with lenvatinib resistance. Cell Death Discov.

