# Supplementary material for: Genome-Wide CRISPR/Cas9 Library Screening Identified that DUSP4 Deficiency Induces Lenvatinib Resistance in Hepatocellular Carcinoma
Source: Int J Biol Sci. 2022 Jul 4;18(11):4357–71. doi: 10.7150/ijbs.69969 (PMC9295068; doi:10.7150/ijbs.69969)

Supplementary materials

Of ***Genome-wide CRISPR/Cas9 library screening identified DUSP4 deficiency induces Lenvatinib resistance in hepatocellular carcinoma***

Figure S1

(A) The expression of Cas9 was confirmed by polymerase chain reaction (PCR). Lane 1 is the marker DL2000. Lane 2, HepG2. Lane 3, HepG2-KO-NC. Lane 4, HepG2-GeCKO A. As shown in the figure, Lane 3 and 4 presented bands of 259bp, indicating the expression of Cas9 in the cells.

A

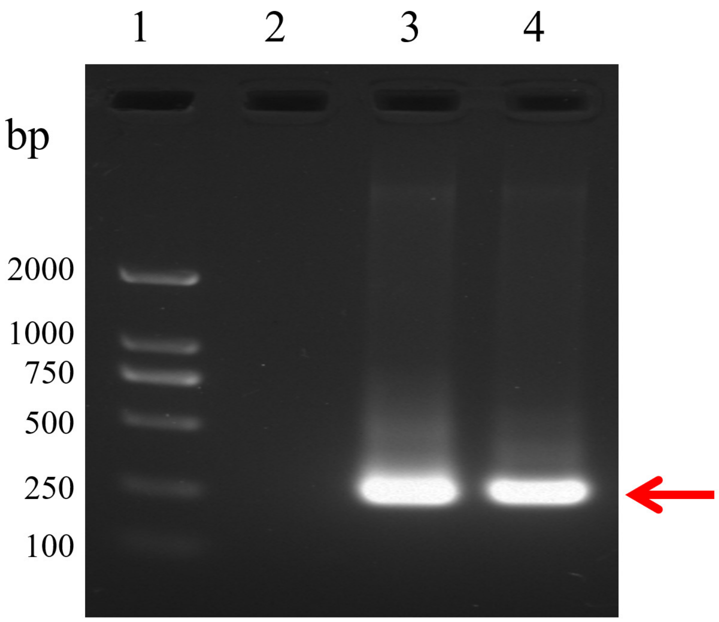

Figure S2

Exploration of minimum lethal concentration of Lenvatinib in HCC cells. **(A)** HepG2 was treated with Lenvatinib at 0, 50 µM, 80 µM,100 µM,120 µM and 140 µM for 72 hours. 80 µM is the minimum lethal concentration of Lenvatinib for HepG2 cells. **(B)** Huh7 was treated with Lenvatinib at 0, 15µM, 20 µM, 30 µM, 40 µM and 50 µM for 72 hours. 20 µM is the minimum lethal concentration of Lenvatinib for Huh7 cells. **(C)** We developed two LR HCC cell lines by culturing HCC cells with long-term exposure to Lenvatinib. LR cells were acquired by gradually increasing the dosages of Lenvatinib over repeated cell passages (4 months). LR cell lines were successfully constructed when HCC cell lines can tolerate higher doses of Lenvatinib compared to wildtype (WT) cell lines. Those two cell lines were characterized by higher cell viability with Lenvatinib treatment. In the resistant cells, the IC50 of Lenvatinib showed a higher concentration comparing to WT cells.

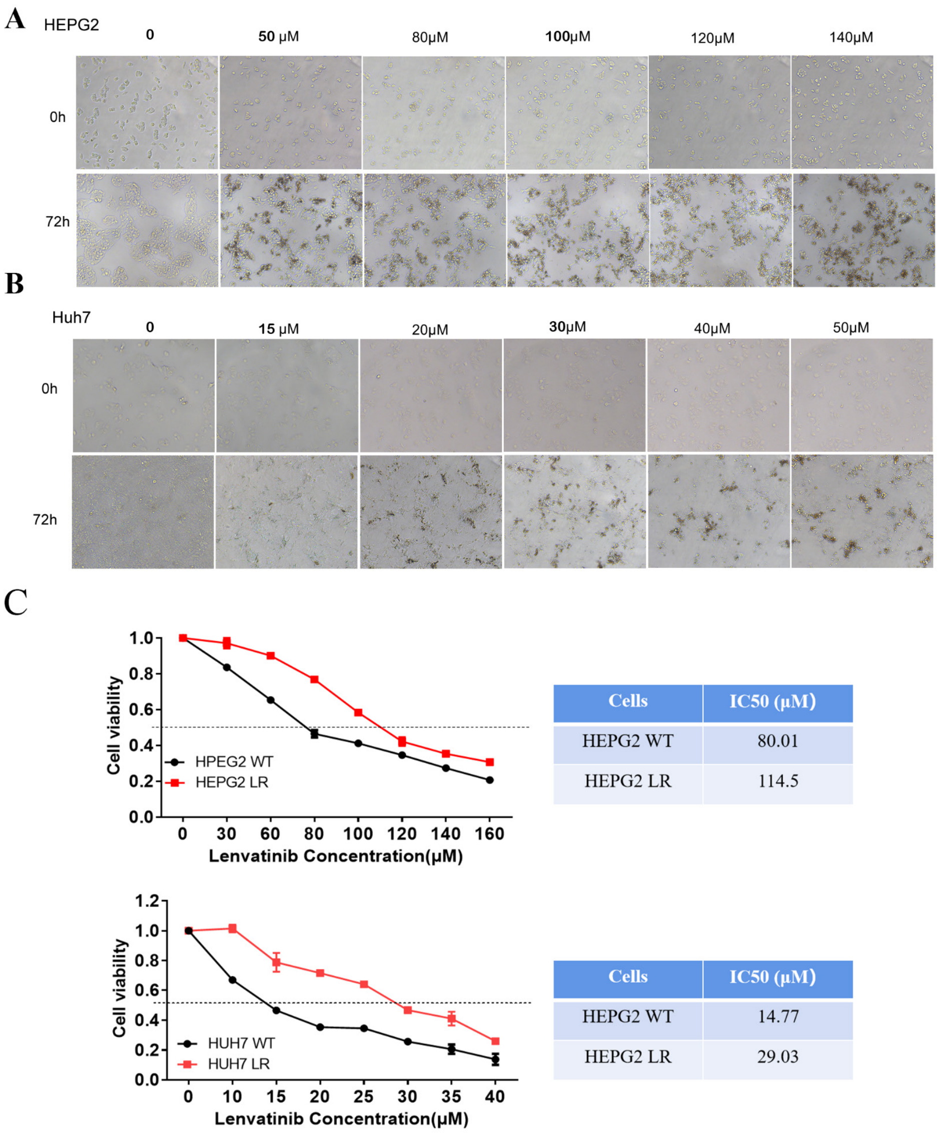

Figure S3

50 µl of 3 x 10^6 HepG2-NC or HepG2-KO library cells were injected into the livers of male BALB/C nude mice aged 6 to 8 weeks in 3 groups, KO-NC, KO-Library and KO- Library + Lenvatinib. There was no significant difference between three groups in the weight of mice.

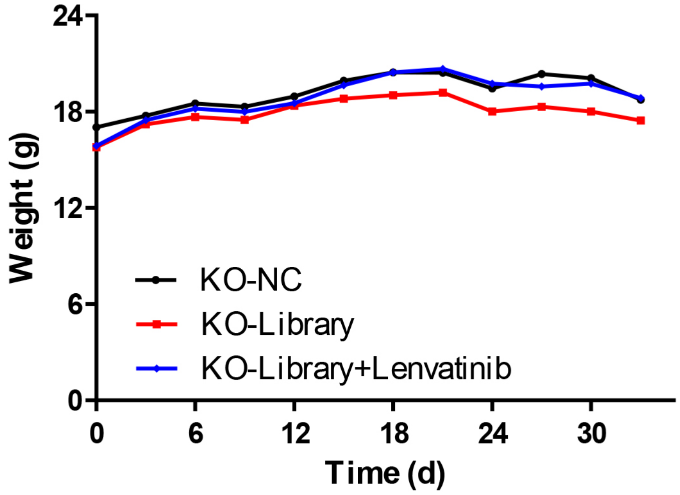

Figure S4

The Lenvatinib resistant model in vivo. **(A)** HCC cells were injected into livers of mice. After 5 weeks, we observed tumors on the livers in all 3 groups, even in Lenvatinib treatment group, indicating Lenvatinib resistance. **(B)** We do not observe metastasis node on the surface of lungs from each group. (C) The HE staining of the liver and lung tissues. No metastasis was observed in KO-NC group. Metastasis were observed in KO-Library groups, even with Lenvatinib treatment. **(D)** The Vimentin staining was weak in KO-NC group, while it was stronger in KO-Library and KO-Library + Lenvatinib groups, especially in lung metastasis.

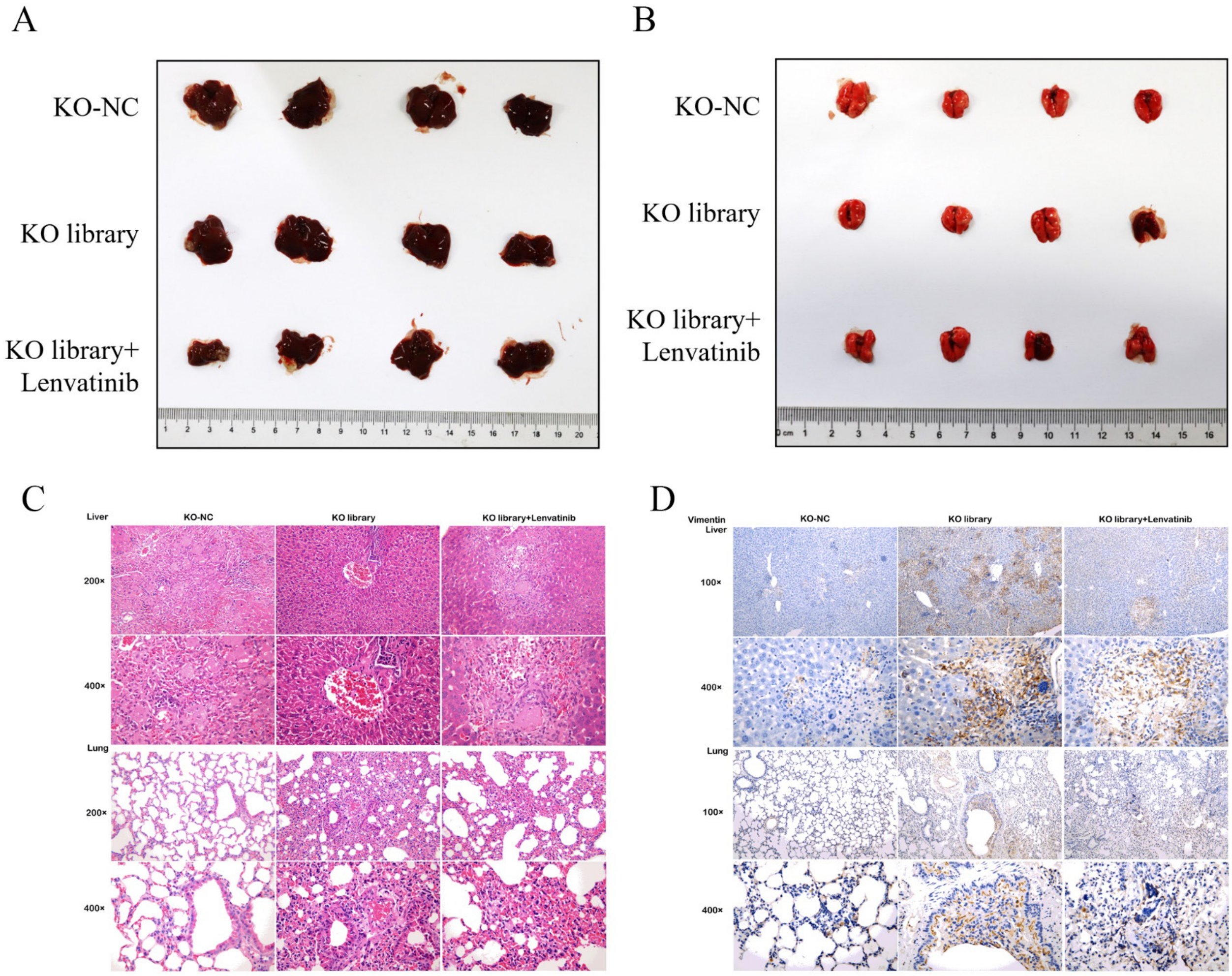

Figure S5: Function enrichment of GO terms by differentially expressed sgRNAs. The most enriched GO terms enriched by differentially expressed sgRNAs from LR vs control cells **(A)**, Primary tumors vs. control cells **(B)**, Lung mets vs. control cells **(C)** and Lung mets vs Primary tumors **(D)**. The cutoff value is $Log_2(FC)>1$ and $p<0.05$.

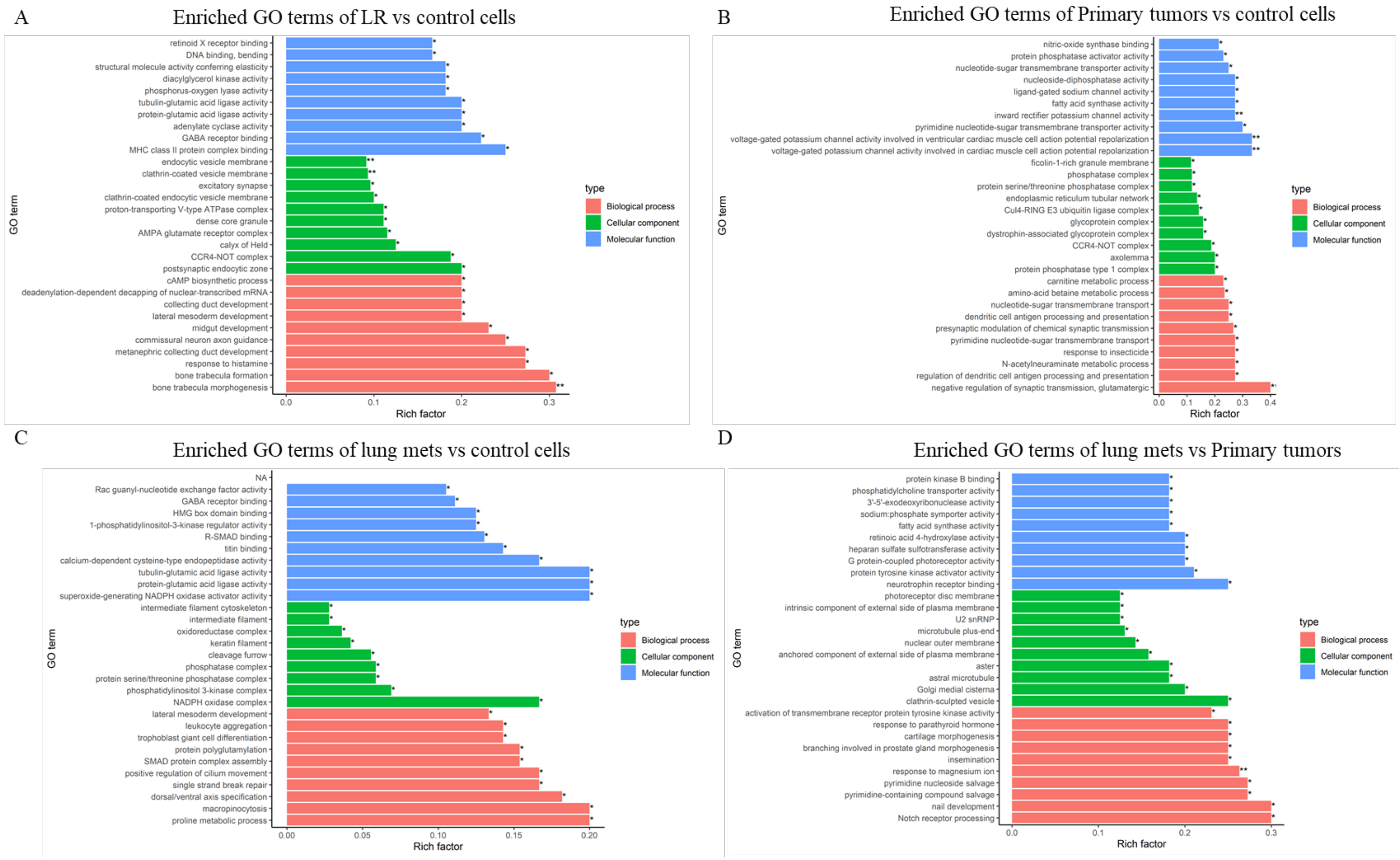

Figure S6: Function enrichment of KEGG pathways by differentially expressed sgRNAs. The most enriched KEGG pathways enriched by differentially expressed sgRNAs from LR vs control cells **(A)**, Primary tumors vs. control cells **(B)**, Lung mets vs. control cells **(C)** and Lung mets vs Primary tumors **(D)**. The cutoff value is $Log_2(FC)>1$ and $p<0.05$.

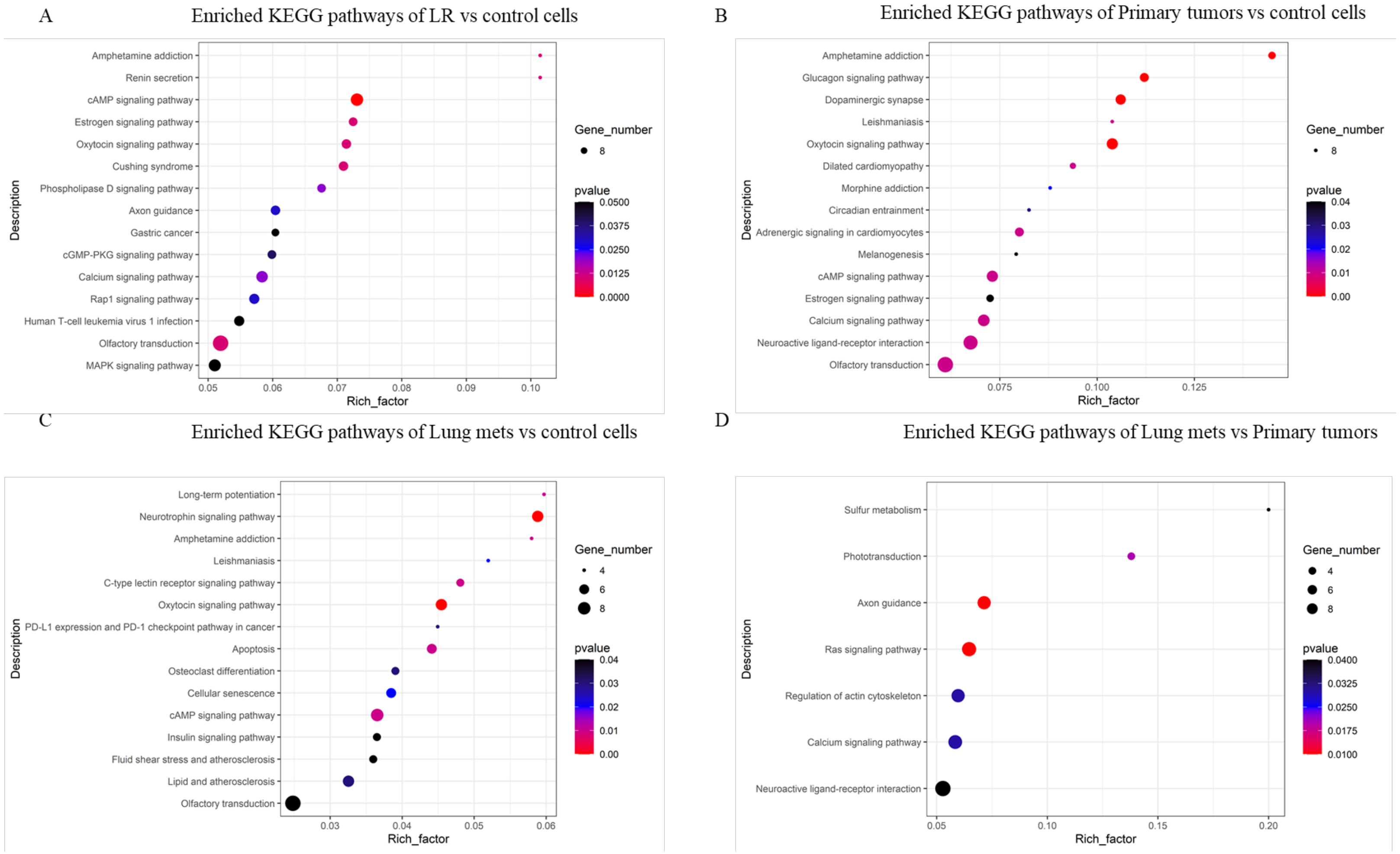

Figure S7: DUSP4 could be a marker for Sorafenib sensitivity. We also investigated whether DUSP4 deficiency was associated with sorafenib resistance by analyzing the expression of DUSP4 in a cohort of HCC patients received sorafenib treatment. **(A)** The data from GSE109211 showed that DUSP4 expression in HCC tissues was comparable between the sorafenib group and the placebo group, while HCC patients that were non-responsive to sorafenib had lower DUSP4 expression levels than responders. **(B)** Meanwhile, high-DUSP4 HCC patients in the course of Sorafenib treatment had a better OS with a very slight trend toward significance (p=0.59) than low-DUSP4 HCC patients. **(C)** Consistently, Hep3B cells that were resistant to Sorafenib had lower levels of DUSP4 as indicated by GSE151412 data.

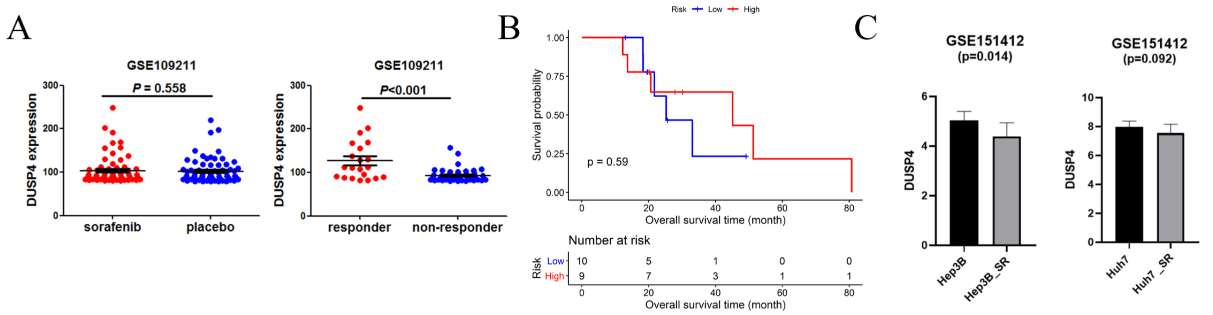

Supplement: Supplementary file 1 — Supplementary figures. [file ijbsv18p4357s1.pdf]
